# Type I Interferon and the Spectrum of Susceptibility to Viral Infection and Autoimmune Disease: A Shared Genomic Signature

**DOI:** 10.3389/fimmu.2021.757249

**Published:** 2021-11-30

**Authors:** Jamie A. Sugrue, Nollaig M. Bourke, Cliona O’Farrelly

**Affiliations:** ^1^ School of Biochemistry and Immunology, Trinity Biomedical Sciences Institute, Trinity College Dublin, Dublin, Ireland; ^2^ Department of Medical Gerontology, School of Medicine, Trinity Translational Medicine Institute, Trinity College Dublin, Dublin, Ireland; ^3^ School of Medicine, Trinity College Dublin, Dublin, Ireland

**Keywords:** infection, autoimmunity, viral resistance, genetics, sexual dimorphism, type I interferons

## Abstract

Type I interferons (IFN-I) and their cognate receptor, the IFNAR1/2 heterodimer, are critical components of the innate immune system in humans. They have been widely explored in the context of viral infection and autoimmune disease where they play key roles in protection against infection or shaping disease pathogenesis. A false dichotomy has emerged in the study of IFN-I where interferons are thought of as either beneficial or pathogenic. This ‘good or bad’ viewpoint excludes more nuanced interpretations of IFN-I biology - for example, it is known that IFN-I is associated with the development of systemic lupus erythematosus, yet is also protective in the context of infectious diseases and contributes to resistance to viral infection. Studies have suggested that a shared transcriptomic signature underpins both potential resistance to viral infection and susceptibility to autoimmune disease. This seems to be particularly evident in females, who exhibit increased viral resistance and increased susceptibility to autoimmune disease. The molecular mechanisms behind such a signature and the role of sex in its determination have yet to be precisely defined. From a genomic perspective, several single nucleotide polymorphisms (SNPs) in the IFN-I pathway have been associated with both infectious and autoimmune disease. While overlap between infection and autoimmunity has been described in the incidence of these SNPs, it has been overlooked in work and discussion to date. Here, we discuss the possible contributions of IFN-Is to the pathogenesis of infectious and autoimmune diseases. We comment on genetic associations between common SNPs in IFN-I or their signalling molecules that point towards roles in protection against viral infection and susceptibility to autoimmunity and propose that a shared transcriptomic and genomic immunological signature may underlie resistance to viral infection and susceptibility to autoimmunity in humans. We believe that defining shared transcriptomic and genomic immunological signatures underlying resistance to viral infection and autoimmunity in humans will reveal new therapeutic targets and improved vaccine strategies, particularly in females.

## Introduction

Type I interferons (IFN-I) are highly conserved key players in innate and adaptive antiviral immune responses. In humans, IFN-I is a multigene family of pleiotropic cytokines comprised of 13 IFNα subtypes, 1 IFNβ, and several other less well defined IFN-Is including IFNε, IFNκ, IFNω (1). IFN-Is are activated by the innate immune system immediately on detection of a threat, particularly when a virus is sensed. This rapid and robust IFN-I immune response which activates and regulates a wide range of biological mechanisms, is required for successful early control of viral infection and is crucial for the activation of long lasting and more specific adaptive immune responses (2). However this ability to activate such pleiotropic biological mechanisms means that IFN-I responses, from their initial activation to their ability to induce downstream signalling, requires tight regulatory control mechanisms. Subtle variations to these responses can have marked physiological effects (3, 4).

**Figure 1:**
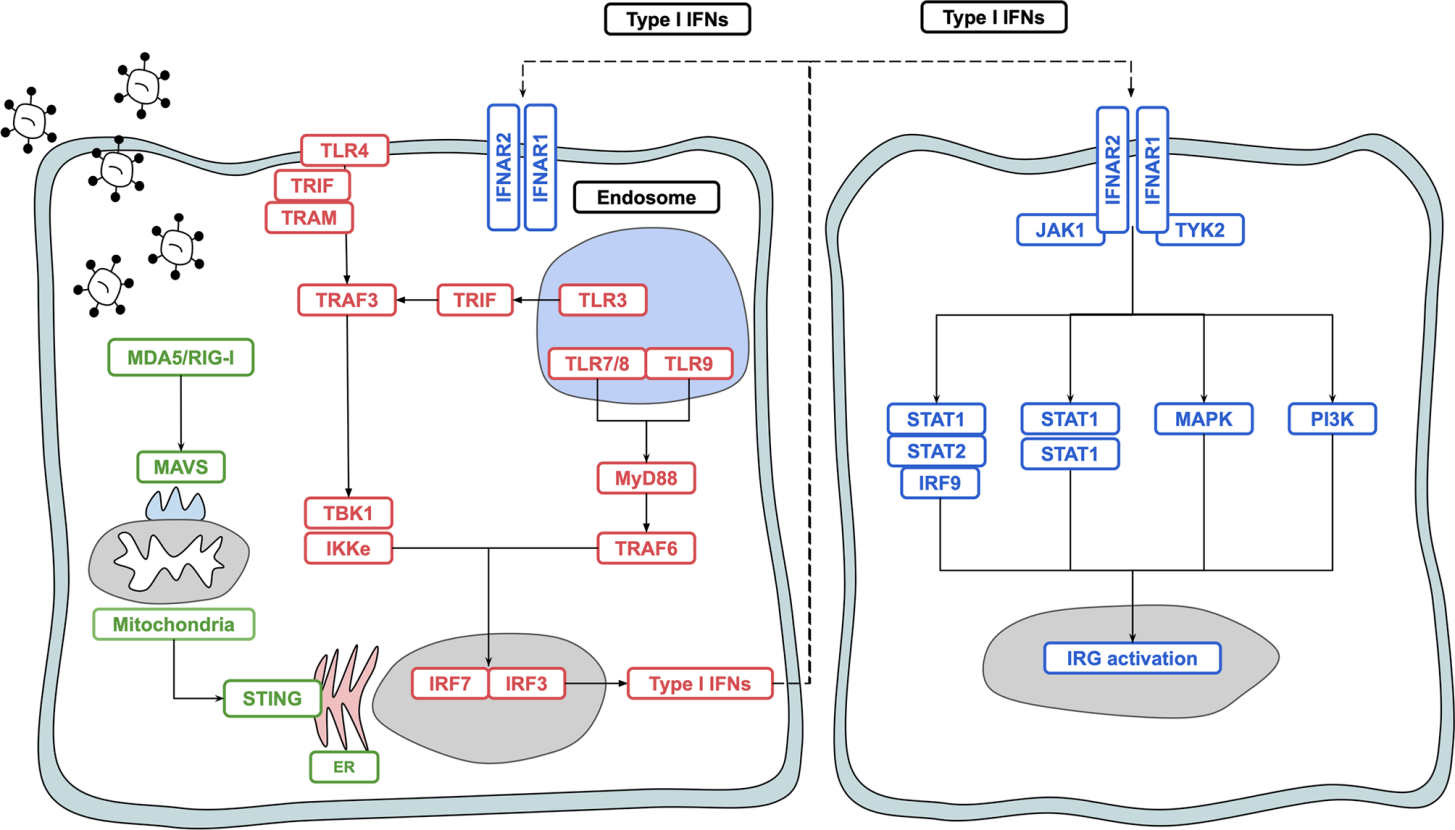
Overview of type I interferon induction and signalling. Activation of PRRs such as TLR4, TLR3, TLR7, TLR8 and RIG-I results in signal transduction and activation of the transcription factors IRF3 and IRF7, leading to production of IFN-I. IFN-I binds to the IFNAR1/IFNAR2 heterodimer and signals in both a paracrine and autocrine manner *via* the JAK/STAT pathway to upregulate interferon regulated genes which act to protect the host against noxious agents such as viruses and bacteria.

IFN-I is produced in response to ligation of pattern recognition receptors (PRRs) including the toll like receptors (TLRs) 3, 7/8 and 9, and the DNA/RNA sensors RIG-I, MDA5 and cGAS-STING (5). These pathways converge to activate the interferon regulatory factor (IRF) transcription factors, chiefly IRF3 and IRF7 (6). Binding of dsRNA to TLR3 results in the activation of a downstream signalling pathway involving the adaptor proteins TRIF and TRAF, which activate TANK binding kinase 1 (TBK1) and IκB Kinase-e (IKKε) activity to phosphorylate and activate the transcription factor IRF3 (7). Activation of IRF7 can also occur and is required for robust IFN-I production (8). While IRF3 is expressed at high levels in homeostatic conditions, IRF7 is more lowly expressed, and is induced following ligation of the TLRs 7,8 and 9 through MyD88 signalling (9, 10). IFN-I also activates IRF7, particularly in pDCs; IRF3 is essential for upregulation of IFN-I genes during the early stages of infection and for potentiating the overall IFN-I response *via* positive-feedback with IRF7. Initial events result in upregulation of IFNβ and IFNα4, which act in a positive feedback loop to upregulate additional IFNα subtypes and interferon regulated genes (IRGs) *via* IRF7 (**Figure 1**) (9).

Plasmacytoid dendritic cells (pDCs), which are found in most tissues of the human body, are the most potent producers of IFN-I (11, 12). Other cell types in the human body are also capable of producing IFN-I, including lymphoid populations and non-immune cells such as epithelial cells, fibroblasts and neurons (13, 14). Canonical IFN-I signalling occurs *via* a heterodimeric complex composed of IFNAR1 and IFNAR2 and expressed on most nucleated cells in the human body. Ligation of the receptor complex activates the JAK-STAT pathway, which in turn acts to upregulate 1000s of IRGs (13, 15, 16). Other pathways activated *via* IFNAR1/2 ligation include the MAPK and PI3K pathways, which leads to a broader range of effects yet to be fully elucidated and discussed elsewhere (2, 17). IFN-Is are also critical in shaping the metabolic shift required to mount a successful immune response (18). IFN-I and IRGs are tightly regulated by negative regulators including ISG15, USP18 and SOCS proteins which act to switch off IFN-I signalling (3). While IRG induction is important for protection against viral infection as well as certain bacterial and protozoan infections, uncontrolled, or inappropriate IRG activation can lead to the development of several autoimmune disease states (2, 19, 20).

Here we briefly outline the roles of IFN-I in viral infection and autoimmunity and point towards variation in the genes that code for components of the IFN-I system that is associated with both protection against viral infection and susceptibility to autoimmunity. We propose that these genetic variants contribute to a shared transcriptomic and genomic signature that may underlie resistance to viral infection and autoimmunity in humans.

## IFN-I in Viral Infection

During viral infection, IFN-I exerts both antiproliferative and cytotoxic effects on cells in order to limit viral replication. While it appears that different viruses can upregulate various modules of IRGs, activation of IFN-Is and subsequent signalling appears to be largely similar (5). Following viral exposure, pattern recognition receptors are activated by double stranded RNA, single stranded RNA, and DNA. Ligation of these receptors triggers a signalling cascade which culminates in the upregulation of IFN-Is followed by IRGs (5). IRGs can act directly or indirectly and at multiple levels to disrupt the viral life cycle and inhibit viral entry. Several viruses have evolved mechanisms to directly subvert the induction and activity of IRGs, a clear indicator of their importance in impeding viral replication (21).

IFN-I also induces survival and maturation of dendritic cells to enhance antigen presentation and upregulates costimulatory molecules including CD40, CD80, CD86. These cells and molecules act in concert to control viral infection (2). IFN-I derived from pDCs forms part of an important T and B lymphocyte axis that is key to an adaptive immune response and antibody production (22). Excessive IFN-I can be detrimental and inhibit or blunt an appropriate antibody response; mechanisms underpinning these observations have yet to be elucidated (2, 23).

As illustrated by the COVID-19 pandemic, the response to viral infection is heterogenous (24). Some individuals, despite lacking typical risk factors, are susceptible to severe disease. Conversely, despite exposure to a high viral load, some individuals appear to be naturally resistant to SARS-CoV-2 infection (25). Virus resistant people such as these have been described in other viral infections, including HIV and HCV (26, 27). While the focus has historically been on immunological susceptibility to viral disease, there is growing interest in viral protection and resistance. Studying resistant individuals may shed light on new pan or virus specific antiviral mechanisms and better vaccines as well as providing tools for identifying individuals who might be protected during future epidemics (28). Studies of HCV resistant individuals have found increased IFN-I in the serum compared to virus susceptible study participants (29). This increased IFN-I could contribute to the viral resistance seen in people who remain uninfected even after viral exposure and could be indicative of heightened or enhanced immune states as described in healthy individuals elsewhere (22).

## IFN-I in Autoimmunity

Autoimmune conditions are diverse and heterogenous disease states that result from an immune response directed against self-antigens. IFN-I is known to play an important role in several of these conditions, including coeliac disease, type I diabetes mellitus (TIDM), systemic lupus erythematosus (SLE) and primary Sjogren’s syndrome (pSS) (30).

Evidence for a causative role of IFN-I in autoimmune disease comes from studies describing excessive IFN-I response following viral infection leading to autoimmune-like conditions (31, 32). People treated with pegylated-IFNα as a therapy for viral infection or melanoma develop a disease that phenocopies ‘naturally occurring’ SLE and coeliac disease (33). Several studies have reported increased IFNα protein levels in the sera of SLE patients. Elevated IFNα protein is accompanied by an increase in an IRG signature score in circulating immune cells and IFN-I regulated protein expression in serum, both of which correlate well with clinically defined disease severity (33). Adding to evidence for IFN-I in autoimmune disease, recent phase III trials using an IFNAR1 monoclonal antibody blocking IFN-I activity (Anifrolumab) to treat SLE shows promising disease modifying activity (34). IFNα protein is also increased in pSS and has an increased IFN-I signature. Taken together, these observations suggest a common IFN-I mediated mechanism in autoimmune disease (35). Similar to viral infection, susceptibility to autoimmunity is variable, and the disease course of individuals with autoimmune disease is often heterogeneous (36).

## A Shared Transcriptomic Signature in Infection and Autoimmunity

Recent evidence suggests that an IFN-I transcriptional signature predictive of a response to vaccination, is the same as has been described to be predictive of flare severity in SLE, a well-documented IFN-I mediated autoimmune disease (22). This signature can predict high and low vaccine responders, is linked to SLE flare intensity, and is centred around a pDC IFN-I T/B lymphocytes axis. The set point score of this axis higher in some healthy individuals, suggesting an increased basal IFN-I. Individuals who had a more highly active axis tended to have increased antibody responses to yellow fever and influenza vaccination. One could hypothetically extend the vaccine findings here to natural infection and imagine a situation in which individuals who have a higher set point score usually only seen following immune challenge would be more resistant to viral infection (22). The authors were unable to explain why this set point score was higher in some individuals in the absence of antigenic stimulation.

Multiple studies have shown that genetics accounts for most interindividual variation in the innate immune response, of which IFN-I is a key player (37, 38). In studies of viral resistance as well as in Tsang’s study the mechanisms underpinning viral resistance, enhanced vaccine responses and SLE autoimmune flares have yet to be elucidated. We suggest that shared gene variants in IFN-I responses, both in relation to their initial induction and also variants related to how they activate their responses, play a dual role in both phenotypes.

## Genetic Variation in Type I Interferon Related Genes Associated With Viral Infection and Autoimmunity

Both autoimmune and infectious disease have strong immunogenetic associations. Historical focus of genetic influences on enhanced resistance to infection has been on MHC variability and monogenic variants, including CCR5delta in HIV, FUT2 in norovirus and DARC in malaria. Monogenic variants resulting in primary immunodeficiencies that increase susceptibility to viral infections include IRF loss of function mutations seen in influenza, herpes simplex encephalitis and COVID-19 (39–47). In the context of autoimmunity, monogenic variants have also been described and include IFIH1 and DNASE1 in SLE [reviewed in (48)]. While causative variants have been explored widely in these settings, they are unlikely to have more ambiguous roles as absence or gain of function in genes are deleterious and often incompatible with a normal health span.

Monogenic disease associated variants are typically uncommon. In contrast, sequence differences that arise due to SNPs are commonly found in the genome (49). SNPs are defined as either synonymous, wherein a change in a nucleotide base does not result in an alteration in the amino acid composition of a protein (codon degeneracy), or non-synonymous (missense), where the amino acid sequence of a protein is altered. Both types of SNPs can contribute to human health and disease (50, 51). Different selective pressures driven by variable disease burdens across populations has led to substantial variation in the minor allele frequencies (MAFs) of SNPs. Studies of SNPs have provided valuable insight into the roles of specific residues in the function of IFN-I related genes and proteins (52).

Association studies of SNPs typically provide an odds ratio, indicating whether presence or absence of the SNP increases or decreases the risk of developing a particular disease state and by what magnitude. Here we discuss association studies of shared autoimmune and infection related SNPs.

## SNPs in Pattern Recognition Receptors and Signalling Pathways

SNPs in PRRs and the PRR signalling pathway have been associated with infectious and autoimmune disease states (**Table 1**). The rs3775291 SNP in TLR3 has been of particular interest globally given its wide and heterologous associations, including HIV, SLE, type I diabetes and idiopathic pulmonary fibrosis (53–56). This is a non-synonymous variant in which a cytosine is replaced with a thymine, leading to a change in the amino acid in the ectodomain at position 412 from a leucine to a phenylalanine (L412F). C is the major allele, whereas T is the minor. The SNP exhibits substantial population variation – the minor T allele is present in just 3% of Africa donors from the 1000 genomes project, while it is present in 33% of east Asian populations (74).

**Table 1:** List of selected SNPs in the type I interferon system.

Gene	SNP	Alleles (Major/Minor)	Amino Acid Change	Associations	Refs.
TLR3	rs3775291	C>T	Leu412Phe	Resistance to HIV-I infection	Sironi, M. et al. (53)
				Increased risk of SLE development	Laska, M. et al. (54)
				Increased risk of type I diabetes mellitus	Assman, T.S. et al. (55)
				Increased risk of idiopathic pulmonary fibrosis	O’Dwyer, D.N. et al. (56)
					
IRF3	rs7251	C>G	Thr427Ser	Increased risk of SLE development	Zhang, F. et al. (57)
				Increased clearance of HPV infection	Wang, S.S. et al. (58)
					
IFNAR1	rs2257167	G>C	Val141Leu	Spontaneous resolution of HBV infection	Zhou, J. et al. (59)
				Increase in lung cancer pain	Reyes-Gibby, C.C. et al. (60)
				Increased risk multiple sclerosis	Leyva, L. et al. (61)
				increased risk of female vitiligo	Traks, T. et al. (62)
					
TYK2	rs23004256	C>A	Val362Phe	Increased risk of systemic sclerosis	López-Isac, E. et al. (63)
				Increased risk of Crohn’s disease	Sato, K. et al. (64)
				Reduced risk of SLE	Sigurdsson, S. et al. (65)
				Reduced risk of psoriasis	Enerback, C. et al. (66)
				No impact of tuberculosis risk	Kerner, G. et al. (67)
					
OAS1	rs10774671	A>G	—	Increased resistance to COVID-19 hospitalisation and severe disease	Zhou, S. et al. (68)
				Increased risk multiple sclerosis	O’Brien, M. et al. (69)
				Increased risk of Sjogren’s syndrome	Li, H. et al. (70)
				Increased resistance to West Nile virus	Lim, J.K. et al. (71)
					
TLR9	rs5743836	A>G	—	Increased spontaneous resolution of HCV in females	Fischer, J. et al. (72)
TLR7	rs179008	A>T	Gln11Leu	Increased HIV-I viremia in females	Azar, P. et al. (73)

The TLR3 SNP has also previously been associated with increased resistance to HIV infection in highly exposed seronegative (HESN) intravenous drug users exposed to HIV (53). Sironi et al. genotyped two independent cohorts and found the frequency of individuals carrying at least one phenylalanine allele is significantly higher in HESN individuals compared to a matched controls (53). The SNP has also been associated with SLE risk and more strongly with development of type I diabetes mellitus (54, 55).

Analysis of data from the Genotype Tissue Expression database (GTEX) shows rs3775291 to be a positive expression quantitative trait loci (eQTL) for TLR3 expression, meaning that it increases TLR3 mRNA levels. Functional analysis of peripheral blood mononuclear cells (PBMCs) in the Sironi study showed variant CT and TT donors to have reduced replication of HIV compared to WT CC donors (53). This reduced HIV replication was accompanied by increased immune activation denoted by marked increases in IL6, CCL3 and the activation marker CD69. In response to a TLR3 agonist, donors with the minor allele CT and TT also had increased upregulation of these markers (53).

Additionally, it appears this polymorphism is associated with enhanced general TLR responsiveness, suggesting that tonic signalling through TLR3 may be important for TLR expression levels and subsequent anti-viral activity and IFN-I production (14). The dual autoimmune and viral resistance association is likely underpinned by the increased TLR3 expression and consequent enhanced immune activation.

Rs7251 is a non-synonymous SNP in IRF3 involving a base change from cytosine to guanine leading to an amino acid substitution at the final position of 427 from a threonine (ACC) to a serine (AGC; T427S) (74). G encodes a serine and is the major allele in most populations with an allele frequency ranging from 50% to 67%, except in Africans where it is the minor allele with a frequency of 29%. G is considered to be the derived risk allele, whereas C appears to be the ancestral allele. The SNP is a blood *cis* eQTL for IRF3 and leads to increased IRF3 expression [GTEX data;7 (57)]. The resultant increased basal expression of IRF3 is likely to lead to a “heightened” immune state that, in one context could contribute to resistance to viral infection, while in another could increase the risk of immune dysregulation and development of autoimmune disease. This notion is reflected in previous association studies in which one study linked the CG and CC genotypes with increased persistence of HPV infection – conversely suggesting that the GG genotype may be associated with increased clearance of HPV infection (58). A recent meta-analysis involving 7,212 cases and 13,556 controls found that the G allele is significantly associated with SLE risk, in particular increased risk of developing an SLE associated inflammatory condition called lupus nephritis (57). This link between the G allele and resistance to HPV infection as well as SLE risk supports our hypothesis of a shared genetic signature underlying viral resistance and susceptibility to autoimmunity.

## SNPs in Type I Interferons and the IFNAR1/2 Receptor

Common SNPs with a MAF > 5% in IFN-I genes are rare. An Ensembl search reveals no non-synonymous SNPs with a MAF greater than 5% for most of the IFN-I subtypes. Those IFN-I genes with SNPs at a high enough frequency have not yet been studied for association with susceptibility to viral infection or autoimmunity. We focus therefore on SNPs in components of the IFN-I receptor.

IFN-I signals *via* the IFNAR1 IFNAR2 heterodimer. SNPs in IFNAR1 and IFNAR2 have been associated with several disease states; associations appear to be highly context and disease specific with the same SNP reported to have both positive and negative effects on disease outcome depending on whether the disease is due to viral infection or autoimmunity. For example, rs2257167 in IFNAR1 has been associated with both spontaneous resolution of hepatitis B virus infection, resistance to respiratory virus infection and increased pain in lung cancer and risk of developing multiple sclerosis and vitiligo in females (59–62, 75). This dual association with both autoimmune susceptibility and protection from viral infections is interesting as it shows that a SNP which is thought to pathogenic in one instance can be protective in another. Rs2257167 results in a valine to leucine substitution at position 141 in the SD2 domain of AR1 (59). As with several other SNPs, the MAF varies dramatically between populations, suggesting a functional consequence driven by different evolutionary pressures (74). While investigations into its functional impact are lacking, limited reports suggest that rs2257167 increases IFNAR1 expression, which may positively impact the antiviral immune response during infection, but also increase the propensity to develop autoimmune disease (59).

## SNPs in Type I Interferon Signalling and Interferon Response Genes

Binding of IFN-I molecules to their receptor leads to phosphorylation of the accessory protein TYK2 and activation of the JAK-STAT pathway (**Figure 1**). Rs2304256 is a SNP found in exon 8 of the TYK2 gene that results in a valine to phenylalanine substitution at position 362 (Val362Phe). 362 is located in the FERM domain (F = 4.1 protein, E = ezrin, R = radixin and M = moesin) of TYK2 which mediates the interaction between the protein and IFNAR1 (63). While this SNP has yet to be explored in the context of viral infection, several studies have investigated its association with autoimmune disease.

Association studies involving the TYK2 SNP are confounding; the minor allele appears to be either protective or deleterious depending on the autoimmune disease in question, underlying the fact that within the umbrella term of autoimmunity, disease states are more nuanced. The variant A allele has been associated with an increased risk of systemic sclerosis and Crohn’s disease (63, 64). However, other work suggests that the SNP may be protective against SLE and psoriasis (65, 66). The SNP is not associated with tuberculosis susceptibility (67).

The variant minor allele is associated with a modest increase in TYK2 expression in whole blood at baseline (76). This could enable increased tonic signalling and greater expression of IFN-I negative regulators leading to a heightened activation threshold and therefore reduced susceptibility to some autoimmune disease. Further studies are warranted to understand the functional impact of the TYK2 SNP on the immune response and to identify potential associations with specific viral infections.

IFN-I signalling culminates in the upregulation of IRGs such as 2-5 oligoadenylate synthase 1 (OAS1), a protein used to synthesize 2’-5- oligoadenylates that activates latent RNaseL, which in turn induces the degradation of viral RNA and inhibits viral replication. There are two major splice variants of OAS1, p42 and p46, each with differing antiviral activity. A splice QTL (sQTL), rs10774671, which increases the expression of the p46 isoform, has been associated with resistance to viral infections such as SARS-CoV-2 and West Nile virus, and also associated with autoimmune diseases including multiple sclerosis and Sjogren’s syndrome (68–71).

G is the minor allele of the OAS1 SNP in the European population and A is the major. The G allele appears to have been reintroduced to the European population through adaptive introgression from Neandertals that was likely driven by flavivirus burden (77, 78). The G allele has been identified as a resistance allele for West Nile virus (71). Both the heterozygote GA and the homozygote GG are associated with increased resistance to HCV infection (79, 80). From an autoimmune perspective, rs10774671 is associated with several autoimmune diseases including type I diabetes, multiple sclerosis and Sjogren’s syndrome (69, 70, 81, 82).

The rs10774671 sQTL increases expression of the p46 isoform which has greater enzymatic activity (70). The increased OAS1 expression is evident in the GTEX whole blood data set and in recent work showing increased localisation of the p46 isoform to the Golgi membrane, which allows for enhanced detection of viral RNA and increased antiviral activity against positive strand RNA viruses that replicate nearby (83). Again, evidence reviewed here indicates a shared genomic signature underlying viral resistance and increased susceptibility to autoimmune diseases.

## Natural Selection, Viral Resistance and Autoimmune Disease Risk

Infectious diseases have a major impact on population mortality; as a consequence, gene variants associated with protection against infection are some of the biggest targets of natural selection in humans (84). This is particularly evident in innate immune genes encoding proteins that form the first line of defence against infection (85).

The prevalence of SNPs in the IFN-I pathway associated with both autoimmune diseases and viral resistance discussed throughout this review varies widely between populations. In part, this variation is likely driven by differences in regional pathogen pressures, wherein SNPs protective against infection are selected for, with a contaminant increase in autoimmune disease risk as a by-product of enhanced immunity against noxious agents. This notion has been reviewed in greater detail by Quintana-Murci and colleagues (86).

With respect to SNPs discussed previously in this review, the prevalence of the C allele of the IFNAR1 SNP rs2257167 reaches 38% in East Asian populations, while it is just 16% in African populations - this variation in positive selection for the C allele could be explained by differences in disease burdens between populations (74). Rs2257167 appears to increase IFNAR1 expression and is associated with increased protection against viral infection (59). Africa has the highest incidence of tuberculosis in the world (87). A potent IFN-I response can hamper control of tuberculosis and so positive selection for alleles that reduce the IFN-I response may be of benefit in regions with high tuberculosis burden (88).

Autoimmune diseases impinge lightly on the ability of humans to successfully bear and raise offspring; genetic variation that increases susceptibility to autoimmune disease while enhancing protection against infection has therefore not been selected against. The negative influences of previously advantageous variation are only being detected since general health in human populations has improved and we have developed the ability to control infectious disease. With continued improvement in human health, healthcare and immunotherapeutic strategies, rare variants in the IFN-I pathway that are protective against autoimmune diseases such as type I diabetes, including IFIH1 rs35337543, are unlikely to become more common (89).

## Sex and SNPs in the Type I Interferon Pathway

Of note, rates of viral resistance and spontaneous clearance of infection are higher in females than in males. Incidence of autoimmune diseases such as multiple sclerosis, SLE and rheumatoid arthritis are also higher in females (90). Indeed, the transcriptomic signature described by Tsang et al. that is predictive of vaccine responses and autoimmune disease flares is also higher in females (22). Despite well-described physiological sexually dimorphic disease associations, studies often fail to appropriately address sex differences. Genome wide association studies analysed by sex have uncovered sex specific SNP associations (91).

Differences in MAFs are unlikely to account for sex specific SNP associations as no large sex differences in SNP MAFs have been described, rather it has been proposed that dimorphism in genotype effects exists between sexes (92). This is evinced in a study by Fischer et al. wherein they describe a female specific association between a SNP in the TLR9 promoter region (rs5743836) and spontaneous clearance of HCV infection (72). TLR9 is important in the detection of viral DNA and upregulation of IFN-I. This SNP maps close to an area in the promoter region coregulated by the transcription factors NFκB and the estrogen receptor alpha (ERα), suggesting that the association differences observed could be due to differential regulation by the female sex hormone and hormone response elements within the TLR9 gene (72).

Functional work on whole blood stimulated with ERα activators on WT and variant female donors showed homozygous WT donors downregulated TLR9 within 3 hours following treatment, whereas in the heterozygotes and homozygous variant donors the downregulation was significantly lower. Negative regulation of TLR9 by oestrogen could explain the attenuation of autoimmune disease often observed during pregnancy when high oestrogen levels are maintained. High oestrogen and a reduction in TLR9 expression during pregnancy could also help explain the increased susceptibility to viral infection during pregnancy (93). Indeed, PBMCs from pregnant women stimulated with HRV43, a human rhinovirus showed significantly reduced IFNα production compared with non-pregnant women (93). IFNa also appears to be positively regulated by female sex hormones (94).

As females have two X chromosomes, and males have only one X and one Y, the second X chromosome in females is transcriptionally silenced so as to achieve dosage compensation between the sexes (95). Several immune genes are found on the X chromosome and are not silenced, therefore a further likely contributor to sexually dimorphic effects of SNPs is escape of X chromosome inactivation in females. TLR7, a PRR involved in the IFN-I response and detection of ssRNA, is one such example; immune cells from females express higher levels of TLR7 and produce more IFNα as a consequence (96). Non-synonymous SNPs in the TLR7 gene therefore likely exert different effects between sexes. Indeed, this phenomenon has been described for the rs179008 SNP which appears not to impact on IFNα production in response to R848 stimulation in males, yet reduces IFNα levels in females (73). A key point to note is that sex differences described in humans may not be present in non-human mammals and caution is required when attempting to dissect sex differences in animal models and extrapolate findings to humans (97). Further explorations of SNPs in the IFN-I pathway and their associations with autoimmune and infectious disease ought to consider males and females separately in order to appropriately discern potential sex effects.

## Conclusions

We propose that similar IFN-I mechanisms contribute to resistance to viral infection and susceptibility to autoimmune disease. Using data from multiple SNP association studies we present evidence of a shared genomic signature in the IFN-I pathway that underlies susceptibility to both conditions. We also highlight potential sex specific effects of SNPs and indicate the importance of including sex in association studies. As research in the field of viral immunology begins to look beyond increased susceptibility to infection and shifts towards understanding viral resistance, shared loci of infection and autoimmunity highlighted here may prove to be useful primers for these studies. IFN-I production is a common pathway feature the two clinical states and is deserving of continued therapeutic focus.

## Author Contributions

JS wrote the manuscript, created the figure, and drafted the table. NB reviewed and edited the manuscript. CO’F supervised the writing and reviewed and edited the manuscript. All authors contributed to the article and approved the submitted version.

## Funding

This work was funded by awards to CO’F through a Science Foundation Ireland Investigator Award (12/IA/1667) and under the Science Foundation Ireland Phase 2 COVID-19 Rapid Response Call (20/COV/8487).

## Conflict of Interest

The authors declare that the research was conducted in the absence of any commercial or financial relationships that could be construed as a potential conflict of interest.

## Publisher’s Note

All claims expressed in this article are solely those of the authors and do not necessarily represent those of their affiliated organizations, or those of the publisher, the editors and the reviewers. Any product that may be evaluated in this article, or claim that may be made by its manufacturer, is not guaranteed or endorsed by the publisher.

## References

[1] Lazear HM Schoggins JW Diamond MS. Shared and Distinct Functions of Type I and Type III Interferons. Immunity (2019) 50:907–23. doi: 10.1016/j.immuni.2019.03.025 PMC6839410 30995506

[2] McNab F Mayer-Barber K Sher A Wack A O’Garra A. Type I Interferons in Infectious Disease. Nat Rev Immunol (2015) 15:87. doi: 10.1038/nri3787 25614319 PMC7162685

[3] Porritt RA Hertzog PJ. Dynamic Control of Type I IFN Signalling by an Integrated Network of Negative Regulators. Trends Immunol (2015) 36:150–60. doi: 10.1016/j.it.2015.02.002 25725583

[4] Rodero MP Decalf J Bondet V Hunt D Rice GI Werneke S. Detection of Interferon Alpha Protein Reveals Differential Levels and Cellular Sources in Disease. J Exp Med (2017) 214:1547–55. doi: 10.1084/jem.20161451 PMC5413335 28420733

[5] Blasius AL Beutler B. Intracellular Toll-Like Receptors. Immunity (2010) 32:305–15. doi: 10.1016/j.immuni.2010.03.012 20346772

[6] Honda K Takaoka A Taniguchi T. Type I Interferon [Corrected] Gene Induction by the Interferon Regulatory Factor Family of Transcription Factors. Immunity (2006) 25:349–60. doi: 10.1016/j.immuni.2006.08.009 16979567

[7] Liu S Cai X Wu J Cong Q Chen X Li T. Phosphorylation of Innate Immune Adaptor Proteins MAVS, STING, and TRIF Induces IRF3 Activation. Science (2015) 347:aaa2630. doi: 10.1126/science.aaa2630 25636800

[8] Honda K Yanai H Takaoka A Taniguchi T. Regulation of the Type I IFN Induction: A Current View. Int Immunol (2005) 17:1367–78. doi: 10.1093/intimm/dxh318 16214811

[9] Marié I Durbin JE Levy DE. Differential Viral Induction of Distinct Interferon-Alpha Genes by Positive Feedback Through Interferon Regulatory Factor-7. EMBO J (1998) 17:6660–9. doi: 10.1093/emboj/17.22.6660 PMC1171011 9822609

[10] Sato M Suemori H Hata N Asagiri M Ogasawara K Nakao K. Distinct and Essential Roles of Transcription Factors IRF-3 and IRF-7 in Response to Viruses for IFN-Alpha/Beta Gene Induction. Immunity (2000) 13:539–48. doi: 10.1016/S1074-7613(00)00053-4 11070172

[11] Wilson NS El-Sukkari D Belz GT Smith CM Steptoe RJ Heath WR. Most Lymphoid Organ Dendritic Cell Types are Phenotypically and Functionally Immature. Blood (2003) 102:2187–94. doi: 10.1182/blood-2003-02-0513 12791652

[12] Asselin-Paturel C Trinchieri G. Production of Type I Interferons: Plasmacytoid Dendritic Cells and Beyond. J Exp Med (2005) 202:461–5. doi: 10.1084/jem.20051395 PMC2212850 16103406

[13] Ali S Mann-Nüttel R Schulze A Richter L Alferink J Scheu S. Sources of Type I Interferons in Infectious Immunity: Plasmacytoid Dendritic Cells Not Always in the Driver’s Seat. Front Immunol (2019) 10:778. doi: 10.3389/fimmu.2019.00778 31031767 PMC6473462

[14] Gao D Ciancanelli MJ Zhang P Harschnitz O Bondet V Hasek M. TLR3 Controls Constitutive IFN-β Antiviral Immunity in Human Fibroblasts and Cortical Neurons. J Clin Invest (2021) 131(1):e134529. doi: 10.1172/JCI134529 PMC7773389 33393505

[15] Schoggins JW. Interferon-Stimulated Genes: What Do They All do? Annu Rev Virol (2019) 6:567–84. doi: 10.1146/annurev-virology-092818-015756 31283436

[16] Rusinova I Forster S Yu S Kannan A Masse M Cumming H. Interferome V2.0: An Updated Database of Annotated Interferon-Regulated Genes. Nucleic Acids Res (2013) 41:D1040–6. doi: 10.1093/nar/gks1215 PMC3531205 23203888

[17] Kaur S Sassano A Joseph AM Majchrzak-Kita B Eklund EA Verma A. Dual Regulatory Roles of Phosphatidylinositol 3-Kinase in IFN Signaling. J Immunol (2008) 181:7316–23. doi: 10.4049/jimmunol.181.10.7316 PMC2597572 18981154

[18] Wu D Sanin DE Everts B Chen Q Qiu J Buck MD. Type 1 Interferons Induce Changes in Core Metabolism That Are Critical for Immune Function. Immunity (2016) 44:1325–36. doi: 10.1016/j.immuni.2016.06.006 PMC5695232 27332732

[19] Motwani M Pesiridis S Fitzgerald KA. DNA Sensing by the cGAS-STING Pathway in Health and Disease. Nat Rev Genet (2019) 20:657–74. doi: 10.1038/s41576-019-0151-1 31358977

[20] Niewold TB. Type I Interferon in Human Autoimmunity. Front Immunol (2014) 5:306. doi: 10.3389/fimmu.2014.00306 25071767 PMC4074699

[21] Stevenson NJ Bourke NM Ryan EJ Binder M Fanning L Johnston JA. Hepatitis C Virus Targets the Interferon-α JAK/STAT Pathway by Promoting Proteasomal Degradation in Immune Cells and Hepatocytes. FEBS Lett (2013) 587:1571–8. doi: 10.1016/j.febslet.2013.03.041 23587486

[22] Kotliarov Y Sparks R Martins AJ Mulè MP Lu Y Goswami M. Broad Immune Activation Underlies Shared Set Point Signatures for Vaccine Responsiveness in Healthy Individuals and Disease Activity in Patients With Lupus. Nat Med (2020) 26:618–29. doi: 10.1038/s41591-020-0769-8 PMC8392163 32094927

[23] Palacio N Dangi T Chung YR Wang Y Loredo-Varela JL Zhang Z. Early Type I IFN Blockade Improves the Efficacy of Viral Vaccines. J Exp Med (2020) 217(12):e20191220. doi: 10.1084/jem.20191220 32820330 PMC7953731

[24] Hadjadj J Yatim N Barnabei L Corneau A Boussier J Smith N. Impaired Type I Interferon Activity and Inflammatory Responses in Severe COVID-19 Patients. Sci (80-) (2020) 369:718–24. doi: 10.1126/science.abc6027 PMC7402632 32661059

[25] Casanova J-L Su HC. A Global Effort to Define the Human Genetics of Protective Immunity to SARS-CoV-2 Infection. Cell (2020) 181:1194–9. doi: 10.1016/j.cell.2020.05.016 PMC7218368 32405102

[26] Zhao NQ Vendrame E Ferreira A-M Seiler C Ranganath T Alary M. Natural Killer Cell Phenotype is Altered in HIV-Exposed Seronegative Women. PloS One (2020) 15:e0238347. doi: 10.1371/journal.pone.0238347 32870938 PMC7462289

[27] Shawa IT Felmlee DJ Hegazy D Sheridan DA Cramp ME. Exploration of Potential Mechanisms of Hepatitis C Virus Resistance in Exposed Uninfected Intravenous Drug Users. J Viral Hepat (2017) 24:1082–8. doi: 10.1111/jvh.12720 28475247

[28] Paludan SR Pradeu T Masters SL Mogensen TH. Constitutive Immune Mechanisms: Mediators of Host Defence and Immune Regulation. Nat Rev Immunol (2021) 21:137–50. doi: 10.1038/s41577-020-0391-5 PMC7418297 32782357

[29] Warshow UM Riva A Hegazy D Thurairajah PH Kaminski ER Chokshi S. Cytokine Profiles in High Risk Injection Drug Users Suggests Innate as Opposed to Adaptive Immunity in Apparent Resistance to Hepatitis C Virus Infection. J Viral Hepat (2012) 19:501–8. doi: 10.1111/j.1365-2893.2011.01574.x 22676363

[30] Decout A Katz JD Venkatraman S Ablasser A. The cGAS–STING Pathway as a Therapeutic Target in Inflammatory Diseases. Nat Rev Immunol (2021) 21:548–69. doi: 10.1038/s41577-021-00524-z PMC8029610 33833439

[31] Arkin LM Moon JJ Tran JM Asgari S O’Farrelly C Casanova J-L. From Your Nose to Your Toes: A Review of SARS-CoV-2 Pandemic-Associated Pernio. J Invest Dermatol (2021) 141(12):2791–6. doi: 10.1016/j.jid.2021.05.024 PMC8279931 34561087

[32] Houen G Trier NH. Epstein-Barr Virus and Systemic Autoimmune Diseases. Front Immunol (2021) 11:3334. doi: 10.3389/fimmu.2020.587380 PMC7817975 33488588

[33] Delgado-Vega AM Alarcón-Riquelme ME Kozyrev SV. Genetic Associations in Type I Interferon Related Pathways With Autoimmunity. Arthritis Res Ther (2010) 12 Suppl 1:S2. doi: 10.1186/ar2883 20392289 PMC2991775

[34] Onuora S. Positive Results for Anifrolumab in Phase III SLE Trial. Nat Rev Rheumatol (2020) 16:125. doi: 10.1038/s41584-020-0384-6 32020079

[35] Batten I Robinson MW White A Walsh C Fazekas B Wyse J. Investigation of Type I Interferon Responses in ANCA-Associated Vasculitis. Sci Rep (2021) 11:8272. doi: 10.1038/s41598-021-87760-4 33859290 PMC8050071

[36] Cho JH Feldman M. Heterogeneity of Autoimmune Diseases: Pathophysiologic Insights From Genetics and Implications for New Therapies. Nat Med (2015) 21:730–8. doi: 10.1038/nm.3897 PMC5716342 26121193

[37] Patin E Hasan M Bergstedt J Rouilly V Libri V Urrutia A. Natural Variation in the Parameters of Innate Immune Cells is Preferentially Driven by Genetic Factors. Nat Immunol (2018) 19:645. doi: 10.1038/s41590-018-0105-3 29476184

[38] Piasecka B Duffy D Urrutia A Quach H Patin E Posseme C. Distinctive Roles of Age, Sex, and Genetics in Shaping Transcriptional Variation of Human Immune Responses to Microbial Challenges. Proc Natl Acad Sci (2018) 115:E488–97. doi: 10.1073/pnas.1714765115 PMC5776984 29282317

[39] Dean M Carrington M Winkler C Huttley GA Smith MW Allikmets R. Genetic Restriction of HIV-1 Infection and Progression to AIDS by a Deletion Allele of the CKR5 Structural Gene. Hemophilia Growth and Development Study, Multicenter AIDS Cohort Study, Multicenter Hemophilia Cohort Study, San Francisco City Cohort, ALIVE. Science (1996) 273:1856–62. doi: 10.1126/science.273.5283.1856 8791590

[40] Liu R Paxton WA Choe S Ceradini D Martin SR Horuk R. Homozygous Defect in HIV-1 Coreceptor Accounts for Resistance of Some Multiply-Exposed Individuals to HIV-1 Infection. Cell (1996) 86:367–77. doi: 10.1016/S0092-8674(00)80110-5 8756719

[41] Samson M Libert F Doranz BJ Rucker J Liesnard C Farber CM. Resistance to HIV-1 Infection in Caucasian Individuals Bearing Mutant Alleles of the CCR-5 Chemokine Receptor Gene. Nature (1996) 382:722–5. doi: 10.1038/382722a0 8751444

[42] Lindesmith L Moe C Marionneau S Ruvoen N Jiang X Lindblad L. Human Susceptibility and Resistance to Norwalk Virus Infection. Nat Med (2003) 9:548–53. doi: 10.1038/nm860 12692541

[43] Miller LH Mason SJ Clyde DF McGinniss MH. The Resistance Factor to Plasmodium Vivax in Blacks. The Duffy-Blood-Group Genotype, FyFy. N Engl J Med (1976) 295:302–4. doi: 10.1056/NEJM197608052950602 778616

[44] Zhang Q Bastard P Liu Z Le Pen J Moncada-Velez M Chen J. Inborn Errors of Type I IFN Immunity in Patients With Life-Threatening COVID-19. Sci (80-) (2020) 370:eabd4570. doi: 10.1126/science.abd4570 PMC7857407 32972995

[45] Initiative, C.-19 H. G. Mapping the Human Genetic Architecture of COVID-19. Nature (2021). doi: 10.1038/s41586-021-03767-x PMC8674144 34237774

[46] Andersen LL Mørk N Reinert LS Kofod-Olsen E Narita R Jørgensen SE. Functional IRF3 Deficiency in a Patient With Herpes Simplex Encephalitis. J Exp Med (2015) 212:1371–9. doi: 10.1084/jem.20142274 PMC4548062 26216125

[47] Andreakos E Abel L Vinh DC Kaja E Drolet BA Zhang Q. A Global Effort to Dissect the Human Genetic Basis of Resistance to SARS-CoV-2 Infection. Nat Immunol (2021). doi: 10.1038/s41590-021-01030-z PMC8524403 34667308

[48] Alperin JM Ortiz-Fernández L Sawalha AH. Monogenic Lupus: A Developing Paradigm of Disease. Front Immunol (2018) 9:2496. doi: 10.3389/fimmu.2018.02496 30459768 PMC6232876

[49] Orr N Chanock S. Common Genetic Variation and Human Disease. Adv Genet (2008) 62:1–32. doi: 10.1016/S0065-2660(08)00601-9 19010252

[50] Kimchi-Sarfaty C Oh JM Kim I-W Sauna ZE Calcagno AM Ambudkar SV. A “Silent” Polymorphism in the MDR1 Gene Changes Substrate Specificity. Science (2007) 315:525–8. doi: 10.1126/science.1135308 17185560

[51] Ramensky V Bork P Sunyaev S. Human non-Synonymous SNPs: Server and Survey. Nucleic Acids Res (2002) 30:3894–900. doi: 10.1093/nar/gkf493 PMC137415 12202775

[52] de Weerd NA Vivian JP Lim SS Huang SU-S Hertzog PJ. Structural Integrity With Functional Plasticity: What Type I IFN Receptor Polymorphisms Reveal. J Leukoc Biol (2020) 108:909–24. doi: 10.1002/JLB.2MR0420-152R 33448473

[53] Sironi M Biasin M Cagliani R Forni D De Luca M Saulle I. A Common Polymorphism in TLR3 Confers Natural Resistance to HIV-1 Infection. J Immunol (2012) 188:818–23. doi: 10.4049/jimmunol.1102179 22174453

[54] Laska MJ Troldborg A Hansen B Stengaard-Pedersen K Junker P Nexø BA. Polymorphisms Within Toll-Like Receptors Are Associated With Systemic Lupus Erythematosus in a Cohort of Danish Females. Rheumatol (Oxford) (2014) 53:48–55. doi: 10.1093/rheumatology/ket316 24064706

[55] Assmann TS de A. Brondani L Bauer AC Canani LH Crispim D. Polymorphisms in the TLR3 Gene Are Associated With Risk for Type 1 Diabetes Mellitus. Eur J Endocrinol (2014) 170:519–27. doi: 10.1530/EJE-13-0963 24408902

[56] O’Dwyer DN Armstrong ME Trujillo G Cooke G Keane MP Fallon PG. The Toll-Like Receptor 3 L412F Polymorphism and Disease Progression in Idiopathic Pulmonary Fibrosis. Am J Respir Crit Care Med (2013) 188:1442–50. doi: 10.1164/rccm.201304-0760OC 24070541

[57] Zhang F Wang Y-F Zhang Y Lin Z Cao Y Zhang H. Independent Replication on Genome-Wide Association Study Signals Identifies IRF3 as a Novel Locus for Systemic Lupus Erythematosus. Front Genet (2020) 11:600. doi: 10.3389/fgene.2020.00600 32719713 PMC7348047

[58] Wang SS Bratti MC Rodríguez AC Herrero R Burk RD Porras C. Common Variants in Immune and DNA Repair Genes and Risk for Human Papillomavirus Persistence and Progression to Cervical Cancer. J Infect Dis (2009) 199:20–30. doi: 10.1086/595563 19012493 PMC3690375

[59] Zhou J Huang JD Poon VKM Chen DQ Chan CCS Ng F. Functional Dissection of an IFN-α/β Receptor 1 Promoter Variant That Confers Higher Risk to Chronic Hepatitis B Virus Infection. J Hepatol (2009) 51:322–32. doi: 10.1016/j.jhep.2009.03.020 19501422

[60] Reyes-Gibby CC Spitz MR Yennurajalingam S Swartz M Gu J Wu X. Role of Inflammation Gene Polymorphisms on Pain Severity in Lung Cancer Patients. Cancer Epidemiol Biomarkers Prev Publ Am Assoc Cancer Res Cosponsored Am Soc Prev Oncol (2009) 18:2636–42. doi: 10.1158/1055-9965.EPI-09-0426 PMC2759856 19773451

[61] Leyva L Fernández O Fedetz M Blanco E Fernandez V Oliver B. IFNAR1 and IFNAR2 Polymorphisms Confer Susceptibility to Multiple Sclerosis But Not to Interferon-Beta Treatment Response. J Neuroimmunol (2005) 163(1–2):165–71. 10.1016/j.jneuroim.2005.02.010 15885318

[62] Traks T Karelson M Reimann E Rätsep R Silm H Vasar E. Association Analysis of Class II Cytokine and Receptor Genes in Vitiligo Patients. Hum Immunol (2016) 77:375–81. doi: 10.1016/j.humimm.2015.09.050 26429320

[63] López-Isac E Campillo-Davo D Bossini-Castillo L Guerra SG Assassi S Simeón CP. Influence of TYK2 in Systemic Sclerosis Susceptibility: A New Locus in the IL-12 Pathway. Ann Rheumatol Dis (2016) 75:1521–6. doi: 10.1136/annrheumdis-2015-208154 PMC7228811 26338038

[64] Sato K Shiota M Fukuda S Iwamoto E Machida H Inamine T. Strong Evidence of a Combination Polymorphism of the Tyrosine Kinase 2 Gene and the Signal Transducer and Activator of Transcription 3 Gene as a DNA-Based Biomarker for Susceptibility to Crohn’s Disease in the Japanese Population. J Clin Immunol (2009) 29:815–25. doi: 10.1007/s10875-009-9320-x PMC2788098 19653082

[65] Sigurdsson S Nordmark G Göring HHH Lindroos K Wiman A-C Sturfelt G. Polymorphisms in the Tyrosine Kinase 2 and Interferon Regulatory Factor 5 Genes are Associated With Systemic Lupus Erythematosus. Am J Hum Genet (2005) 76:528–37. doi: 10.1086/428480 PMC1196404 15657875

[66] Enerbäck C Sandin C Lambert S Zawistowski M Stuart PE Verma D. The Psoriasis-Protective TYK2 I684S Variant Impairs IL-12 Stimulated Pstat4 Response in Skin-Homing CD4+ and CD8+ Memory T-Cells. Sci Rep (2018) 8:7043. doi: 10.1038/s41598-018-25282-2 29728633 PMC5935702

[67] Kerner G Ramirez-Alejo N Seeleuthner Y Yang R Ogishi M Cobat A. Homozygosity for TYK2 P1104A Underlies Tuberculosis in About 1% of Patients in a Cohort of European Ancestry. Proc Natl Acad Sci U S A (2019) 116:10430–4. doi: 10.1073/pnas.1903561116 PMC6534977 31068474

[68] Zhou S Butler-Laporte G Nakanishi T Morrison DR Afilalo J Afilalo M. A Neanderthal OAS1 Isoform Protects Individuals of European Ancestry Against COVID-19 Susceptibility and Severity. Nat Med (2021) 27:659–67. doi: 10.1038/s41591-021-01281-1 33633408

[69] O’Brien M Lonergan R Costelloe L O’Rourke K Fletcher JM Kinsella K. OAS1: A Multiple Sclerosis Susceptibility Gene That Influences Disease Severity. Neurology (2010) 75:411–8. doi: 10.1212/WNL.0b013e3181ebdd2b 20679634

[70] Li H Reksten TR Ice JA Kelly JA Adrianto I Rasmussen A. Identification of a Sjögren’s Syndrome Susceptibility Locus at OAS1 That Influences Isoform Switching, Protein Expression, and Responsiveness to Type I Interferons. PloS Genet (2017) 13:e1006820. doi: 10.1212/WNL.0b013e3181ebdd2b 28640813 PMC5501660

[71] Lim JK Lisco A McDermott DH Huynh L Ward JM Johnson B. Genetic Variation in OAS1 is a Risk Factor for Initial Infection With West Nile Virus in Man. PloS Pathog (2009) 5:e1000321. doi: 10.1371/journal.ppat.1000321 19247438 PMC2642680

[72] Fischer J Weber ANR Böhm S Dickhöfer S El Maadidi S Deichsel D. Sex-Specific Effects of TLR9 Promoter Variants on Spontaneous Clearance of HCV Infection. Gut (2017) 66:1829–37. doi: 10.1136/gutjnl-2015-310239 27196570

[73] Azar P Mejía JE Cenac C Shaiykova A Youness A Laffont S. TLR7 Dosage Polymorphism Shapes Interferogenesis and HIV-1 Acute Viremia in Women. JCI Insight (2020) 5(12):e136047. doi: 10.1172/jci.insight.136047 PMC7406249 32554924

[74] Yates AD Achuthan P Akanni W Allen J Allen J Alvarez-Jarreta J. Ensembl 2020. Nucleic Acids Res (2020) 48:D682–8. doi: 10.1093/nar/gkz966 PMC7145704 31691826

[75] Antunes KH Fachi JL de Paula R da Silva EF Pral LP dos Santos AÁ. Microbiota-Derived Acetate Protects Against Respiratory Syncytial Virus Infection Through a GPR43-Type 1 Interferon Response. Nat Commun (2019) 10:3273. doi: 10.1038/s41467-019-11152-6 31332169 PMC6646332

[76] Li Z Rotival M Patin E Michel F Pellegrini S. Two Common Disease-Associated TYK2 Variants Impact Exon Splicing and TYK2 Dosage. PloS One (2020) 15:e0225289. doi: 10.1371/journal.pone.0225289 31961910 PMC6974145

[77] Sams AJ Dumaine A Nédélec Y Yotova V Alfieri C Tanner JE. Adaptively Introgressed Neandertal Haplotype at the OAS Locus Functionally Impacts Innate Immune Responses in Humans. Genome Biol (2016) 17:246. doi: 10.1186/s13059-016-1098-6 27899133 PMC5129249

[78] Enard D Petrov DA. Evidence That RNA Viruses Drove Adaptive Introgression Between Neanderthals and Modern Humans. Cell (2018) 175:360–371.e13. doi: 10.1016/j.cell.2018.08.034 30290142 PMC6176737

[79] Kwon Y-C Kang J-I Hwang SB Ahn B-Y. The Ribonuclease L-Dependent Antiviral Roles of Human 2′,5′-Oligoadenylate Synthetase Family Members Against Hepatitis C Virus. FEBS Lett (2013) 587:156–64. doi: 10.1016/j.febslet.2012.11.010 23196181

[80] El Awady MK Anany MA Esmat G Zayed N Tabll AA Helmy A. Single Nucleotide Polymorphism at Exon 7 Splice Acceptor Site of OAS1 Gene Determines Response of Hepatitis C Virus Patients to Interferon Therapy. J Gastroenterol Hepatol (2011) 26:843–50. doi: 10.1111/j.1440-1746.2010.06605.x PMC7166793 21182542

[81] Qu H-Q Polychronakos C the T. I. D. G. Consortium. Reassessment of the Type I Diabetes Association of the OAS1 Locus. Genes Immun (2009) 10:S69–73. doi: 10.1038/gene.2009.95 PMC2805449 19956105

[82] Cagliani R Fumagalli M Guerini FR Riva S Galimberti D Comi GP. Identification of a New Susceptibility Variant for Multiple Sclerosis in OAS1 by Population Genetics Analysis. Hum Genet (2012) 131:87–97. doi: 10.1007/s00439-011-1053-2 21735172 PMC7088416

[83] Soveg FW Schwerk J Gokhale NS Cerosaletti K Smith JR Pairo-Castineira E. Endomembrane Targeting of Human OAS1 P46 Augments Antiviral Activity. Elife (2021) 10:e71047. doi: 10.7554/eLife.71047 34342578 PMC8357416

[84] Fumagalli M Sironi M Pozzoli U Ferrer-Admettla A Pattini L Nielsen R. Signatures of Environmental Genetic Adaptation Pinpoint Pathogens as the Main Selective Pressure Through Human Evolution. PloS Genet (2011) 7:1–14. doi: 10.1371/annotation/ca428083-dbcb-476a-956c-d7bb6e317cf7 PMC3207877 22072984

[85] Quintana-Murci L Clark AG. Population Genetic Tools for Dissecting Innate Immunity in Humans. Nat Rev Immunol (2013) 13:280–93. doi: 10.1038/nri3421 PMC4015519 23470320

[86] Barreiro LB Quintana-Murci L. From Evolutionary Genetics to Human Immunology: How Selection Shapes Host Defence Genes. Nat Rev Genet (2010) 11:17–30. doi: 10.1038/nrg2698 19953080

[87] Glaziou P Sismanidis C Floyd K Raviglione M. Global Epidemiology of Tuberculosis. Cold Spring Harb Perspect Med (2014) 5:a017798. doi: 10.1101/cshperspect.a017798 25359550 PMC4315920

[88] Zhang G deWeerd NA Stifter SA Liu L Zhou B Wang W. A Proline Deletion in IFNAR1 Impairs IFN-Signaling and Underlies Increased Resistance to Tuberculosis in Humans. Nat Commun (2018) 9:85. doi: 10.1038/s41467-017-02611-z 29311663 PMC5758831

[89] Nejentsev S Walker N Riches D Egholm M Todd JA. Rare Variants of IFIH1, a Gene Implicated in Antiviral Responses, Protect Against Type 1 Diabetes. Science (2009) 324:387–9. doi: 10.1126/science.1167728 PMC2707798 19264985

[90] Ngo ST Steyn FJ McCombe PA. Gender Differences in Autoimmune Disease. Front Neuroendocrinol (2014) 35:347–69. doi: 10.1016/j.yfrne.2014.04.004 24793874

[91] Liu LY Schaub MA Sirota M Butte AJ. Sex Differences in Disease Risk From Reported Genome-Wide Association Study Findings. Hum Genet (2012) 131:353–64. doi: 10.1007/s00439-011-1081-y PMC3260375 21858542

[92] Khramtsova EA Davis LK Stranger BE. The Role of Sex in the Genomics of Human Complex Traits. Nat Rev Genet (2019) 20:173–90. doi: 10.1038/s41576-018-0083-1 30581192

[93] Forbes RL Gibson PG Murphy VE Wark PAB. Impaired Type I and III Interferon Response to Rhinovirus Infection During Pregnancy and Asthma. Thorax (2012) 67:209–14. doi: 10.1136/thoraxjnl-2011-200708 21917654

[94] Griesbeck M Ziegler S Laffont S Smith N Chauveau L Tomezsko P. Sex Differences in Plasmacytoid Dendritic Cell Levels of IRF5 Drive Higher IFN-α Production in Women. J Immunol (2015) 195:5327–36. doi: 10.4049/jimmunol.1501684 PMC4654231 26519527

[95] Avner P Heard E. X-Chromosome Inactivation: Counting, Choice and Initiation. Nat Rev Genet (2001) 2:59–67. doi: 10.1038/35047580 11253071

[96] Souyris M Cenac C Azar P Daviaud D Canivet A Grunenwald S. TLR7 Escapes X Chromosome Inactivation in Immune Cells. Sci Immunol (2018) 3:eaap8855. doi: 10.1126/sciimmunol.aap8855 29374079

[97] Naqvi S Godfrey AK Hughes JF Goodheart ML Mitchell RN Page DC. Conservation, Acquisition, and Functional Impact of Sex-Biased Gene Expression in Mammals. Sci (80-) (2019) 365(6450):eaaw7317. doi: 10.1126/science.aaw7317 PMC6896219 31320509

